# IL-8 Released from Human Pancreatic Cancer and Tumor-Associated Stromal Cells Signals through a CXCR2-ERK1/2 Axis to Induce Muscle Atrophy

**DOI:** 10.3390/cancers11121863

**Published:** 2019-11-25

**Authors:** Chandler S. Callaway, Andrea E. Delitto, Andrew C. D’Lugos, Rohan Patel, Rachel L. Nosacka, Daniel Delitto, Michael R. Deyhle, Jose G. Trevino, Sarah M. Judge, Andrew R. Judge

**Affiliations:** 1 Department of Physical Therapy, University of Florida, Gainesville, FL 32610, USA; chandlercallaway@ufl.edu (C.S.C.); andreak@phhp.ufl.edu (A.E.D.); adlugos@phhp.ufl.edu (A.C.D.); rohan1598741@ufl.edu (R.P.); rnoscaka@ufl.edu (R.L.N.); mdeyhle@phhp.ufl.edu (M.R.D.); smsenf@phhp.ufl.edu (S.M.J.); 2 Department of Surgery, University of Florida, Gainesville, FL 32610, USA; Ddelitt1@jhmi.edu (D.D.); Jose.Trevino@surgery.ufl.edu (J.G.T.)

**Keywords:** muscle wasting, cachexia, interleukin-8

## Abstract

Tumor-derived cytokines are known to drive the catabolism of host tissues, including skeletal muscle. However, our understanding of the specific cytokines that initiate this process remains incomplete. In the current study, we conducted multiplex analyte profiling of cytokines in conditioned medium (CM) collected from human pancreatic cancer (PC) cells, human tumor-associated stromal (TAS) cells, and their co-culture. Of the factors identified, interleukin-8 (IL-8) is released at high levels from PC cells and PC/TAS co-culture and has previously been associated with low muscle mass in cancer patients. We, therefore, treated C2C12 myotubes with IL-8 which led to the activation of ERK1/2, STAT, and Smad signaling, and induced myotube atrophy. Moreover, the treatment of mice with IL-8 also induced significant muscle wasting, confirming the in vivo relevance of IL-8 on muscle. Mechanistically, IL-8-induced myotube atrophy is inhibited by treatment with the CXCR2 antagonist, SB225002, or by treatment with the ERK1/2 inhibitor, U0126. We further demonstrate that this axis mediates muscle atrophy induced by pancreatic cancer cell CM, as neutralization of IL-8 or treatment with SB225002 or U0126 significantly inhibit CM-induced myotube atrophy. Thus, these data support a key role of IL-8 released from human PC cells in initiating atrophy of muscle cells via CXCR2-ERK1/2.

## 1. Introduction

Involuntary body weight loss, or cachexia, is a devastating consequence of many advanced-stage cancers, and cachexia ultimately affects up to 80% of all cancer patients [1]. The body weight loss is due to the loss of skeletal muscle mass, which represents approximately 40% of body weight, with or without the accompanying loss of fat mass [2]. For patients, cachexia has a profound impact on the quality of life as it associates with weakness, functional decline, and loss of independence [3]. Cachexia also represents a major barrier to treatment, such as surgery or chemotherapy, as outcomes are highly dependent on functional reserve [4]. This contributes to a significantly decreased survival time in cachectic cancer patients [5]. Thus, identifying modifiable factors essential to the development of cancer cachexia is imperative.

One consensus in the field is that tumor-derived factors are important mediators of cachexia. Therefore, the most common in vitro muscle model of cancer cachexia is the treatment of cultured skeletal muscle myotubes with media collected from cultured cancer cells (conditioned media (CM)). The most widely used cachexia-inducing cell lines are the mouse colon adenocarcinoma 26 (C26) and Lewis lung carcinoma (LLC) cells [4], and treatment of C2C12 mouse skeletal muscle myotubes with C26 or LLC CM induces significant atrophy [6,7,8]. Specific proteins identified in these CM that mediate cachexia include interleukin-6 (IL-6), leukemia inhibitory factor (LIF), tumor necrosis factor alpha (TNF-α), and heat shock protein 70 (Hsp70) [6,9,10,11,12]. Further, multiple signaling pathways have been implicated in these models of muscle wasting, including STAT3, ERK1/2, NFĸB, FOXO pathways, and, more recently, a TLR/MyD88/XBP1 signaling axis [6,7,8,9,10,11,12]. However, as the field continues to expand into the study of additional cachexia-inducing cancer cell lines, we are likely to learn much more about additional factors and pathways driving cachexia. 

In the current study, we aim to identify factors released from human pancreatic cancer (PC) cells, which may be candidates for driving cachexia. PC has one of the highest incidences of cachexia, present in more than 60% of patients at the time of diagnosis and more than 80% of advanced-stage patients [5]. The decision to use human, rather than mouse, PC cells is based on known differences which exist in some cytokines and chemokines expressed in humans versus mice [13,14,15]. Moreover, in recent years it has become clear that cancer cells interact extensively with the tumor microenvironment, consisting of a variety of host cell types. Of particular relevance in PC is the tumor-associated stromal (TAS) cell, which accounts for up to 80% of the tumor mass in the desmoplastic microenvironment of PC. TAS cells themselves secrete a variety of growth factors and inflammatory mediators [16], and the interactions between TAS cells and cancer cells play an important role in supporting tumor growth and survival [17]. However, to our knowledge, no study has determined the ability of TAS secreted factors to induce myotube atrophy or determined whether conditioned media from TAS/cancer cell co-culture induces a synergistic increase in cytokine/chemokine release and greater atrophy. The current study therefore, first aims to test these unknowns and subsequently use the knowledge gained to test the role of an identified chemokine as a trigger of muscle wasting.

## 2. Results

### 2.1. Conditioned Medium from Human Pancreatic Cancer and Stromal Cells Induces Myotube Atrophy

**Figure 1:**
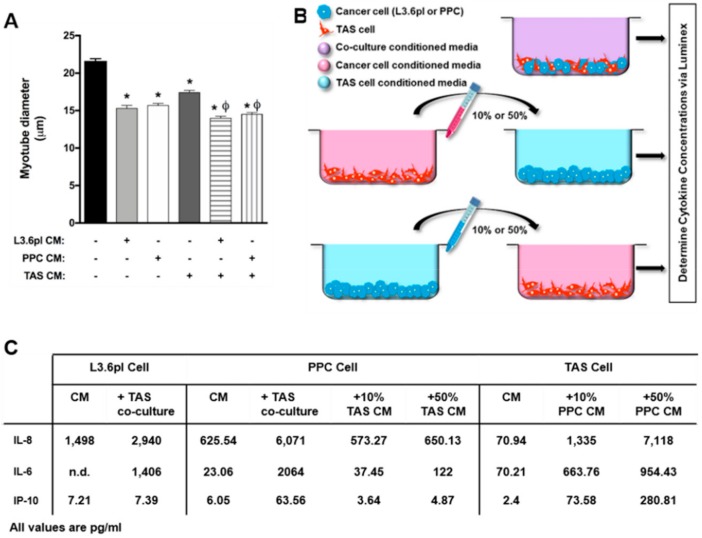
Atrophy in response to, and cytokine concentrations in, conditioned medium (CM) from human pancreatic cancer cells and stromal cells. (**A**) C2C12 myotube diameter following 48 h of treatment with CM from human pancreatic cancer cells (L3.6pl and PPC), human tumor-associated stromal (TAS) cells, or co-culture CM. Data presented as mean ± SEM. * *p* < 0.05 compared with control. ϕ *p* < 0.05 compared to L3.6pl/PPC or TAS CM only. (**B**) Schematic drawing depicting generation of CM by co-culture of L3.6pl or PPC cells with TAS cells, PPC cells with either 10% or 50% TAS CM, or TAS cells stimulated with either 10% or 50% PPC CM for 24 h. (**C**) Concentrations of IL-8, IL-6, and IP-10 (pg/mL) in CM.

Pancreatic tumors are known to be highly cachectogenic, but to first determine the extent to which human pancreatic cancer and stromal cells induce atrophy of muscle cells, in vitro, we collected conditioned media (CM) from L3.6pl human pancreatic cancer cells, primary human pancreatic cancer (PPC) cells, primary human pancreatic tumor-associated stromal cells (TAS) cells, and the co-culture of L3.6pl/TAS and PPC/TAS cells. We then treated myotubes with CM from each, for 48 h, and fixed for the measurement of myotube diameter. As shown in Figure 1A, L3.6pl, PPC, and TAS cell CM induced a 29%, 27%, and 19% decrease in myotube diameter, respectively, whereas L3.6pl/TAS and PC/TAS CM induced a 35% and 33% decrease in myotube diameter, respectively. These findings demonstrate that factors released from human pancreatic cancer and stromal cells induce significant myotube atrophy and that the atrophy induced by cancer/stromal cell interactions is greater than that induced by either cell type alone.

### 2.2. Identification of Cytokines and Chemokines Released from Human Panceratic Cancer Cells and Human Tumor Associated Stromal Cells

To identify cytokines and chemokines secreted from human pancreatic cancer and stromal cells, which might be responsible for the observed myotube atrophy, we conducted multiplex analyte profiling on three pooled samples for each CM. Of the 41 secreted factors analyzed, 28 were detectable in the CM of at least one CM group (Supplementary Table S1). Of these, six were commonly released, at levels >10 pg/ml, from the two different human pancreatic cancer cells. These were epidermal growth factor (EGF), monocyte chemoattractant protein-1/C-C motif chemokine ligand 2 (MCP-1/CCL2), interleukin-8 (IL-8), growth regulated oncogene (GRO), fractalkine, and vascular endothelial growth factor (VEGF). Of these, only IL-8 and GRO were commonly released at levels >500 pg/mL. We similarly profiled CM from primary pancreatic tumor associated stromal (TAS) cells, which secreted very high levels of EGF (4337 pg/mL) and MCP-1/CCL2 (4,951 pg/mL), moderate levels of IL-8 (70.94 pg/mL), and low levels of GRO (18.65 pg/mL). 

We subsequently screened CM from PPC/TAS co-cultures and L3.6pl/TAS co-cultures, as illustrated in Figure 1B, to determine whether the secretion of factors was redundant, additive, or synergistic. Interestingly, the same 5 cytokines were present at high levels in PPC/TAS CM as in the L3.6pl/TAS CM. These were IL-8, IL-6, GRO, MCP-1, and EGF, and for both IL-8 and IL-6, their increase in co-culture CM was synergistic. Indeed, IL-8 levels were 1498 pg/mL in L3.6pl CM, 625.54 pg/mL in PPC cell CM, and 70 pg/mL in TAS CM, but increased to 2940 pg/mL in L3.6pl/TAS cell CM and 6071 pg/mL in PPC/TAS cell CM. Similarly, IL-6 levels were not detectable in L3.6pl CM, were 23.06pg/mL in PPC cell CM, and 70.21 pg/mL in TAS CM, but increased to 1403 pg/mL in L3.6pl/TAS CM and 2064 pg/mL in PPC/TAS CM. Interferon gamma-induced protein 10/C-X-C-motif chemokine ligand 10 (IP-10/CXCL10) also increased synergistically in PPC/TAS CM to 63.56 from 6.05 pg/mL in PPC cell CM and 2.4 pg/mL in TAS CM (Figure 1C). 

These co-culture experiments provide important data regarding the cross-talk between human pancreatic cancer and stromal cells and their release of cytokines. However, for IL-8, IL-6, and IP-10, which show a synergistic increase, the experimental design does not allow us to identify whether stromal cells stimulate their release from cancer cells or cancer cells stimulate their release from stromal cells. To test this, we added TAS CM to PPC cells or PPC cell CM to TAS cells, at a 1:10 or 1:1 ratio for 24 h before collecting the final CM, as illustrated in Figure 1B. The results from these experiments demonstrate that PPC cell CM stimulates the release of IL-8, IL-6, and IP-10 from TAS cells but that TAS cell CM does not stimulate the further release of these cytokines from PPC cells. 

Overall these findings clearly demonstrate the importance of considering cancer and stromal cell interactions when identifying potential tumor-derived cachexia-inducing factors. 

### 2.3. Interleukin-8 is Sufficient to Induce Skeletal Muscle Atrophy

**Figure 2:**
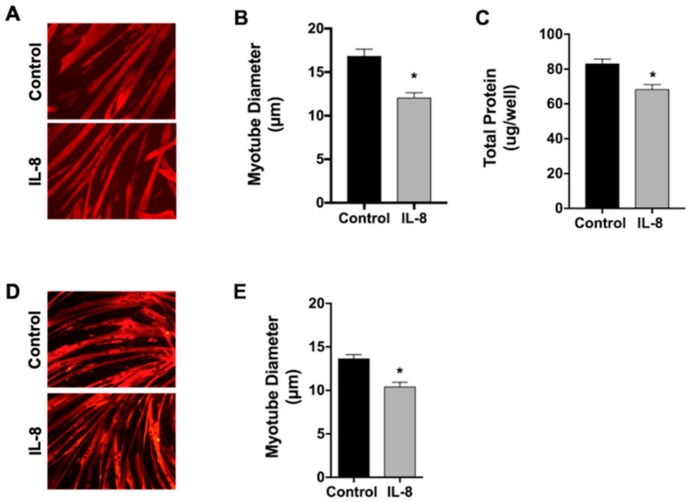
IL-8 induced atrophy of myotubes. C2C12 myotubes (**A**–**C**) and primary human myotubes (**D**,**E**) were treated with 10 ng/mL of recombinant human IL-8, or BSA as a control, for 48 h. Myotubes were immunostained for myosin heavy chain (**A**,**D**) and diameter quantified (**B**,**E**). (**C**) Total protein in control and IL-8 treated C2C12 myotubes. Data presented as mean ± SEM. * *p* < 0.05 compared with control.

Based on these findings, coupled with the knowledge that IL-8 is significantly increased in the serum of cachectic compared to non-cachectic patients with pancreatic, prostate, and gastroesophageal cancers [18,19,20], we elected to focus our subsequent studies on the role of IL-8 in skeletal muscle atrophy. To first test whether an increase in IL-8 is sufficient to cause atrophy of muscle cells, we treated 4-day differentiated C2C12 myotubes with either BSA, as a control, or recombinant IL-8 (rIL-8) for 48 h. As shown in Figure 2A,B, treatment of myotubes with 10ng/ml of rIL-8 caused a 29% decrease in myotube diameter, demonstrating that IL-8 can act directly on muscle cells to induce atrophy. In support of this, IL-8 also causes a significant decrease in the total protein levels of myotubes (Figure 2C). To test whether human myotubes respond to rIL-8 in a manner similar to C2C12 myotubes, we subsequently treated 4-day differentiated human skeletal myotubes with 10ng/ml of rIL-8 for 48 h and, similar to mouse myotubes, this was sufficient to induce a 24% decrease in myotube diameter (Figure 2D,E).

**Figure 3:**
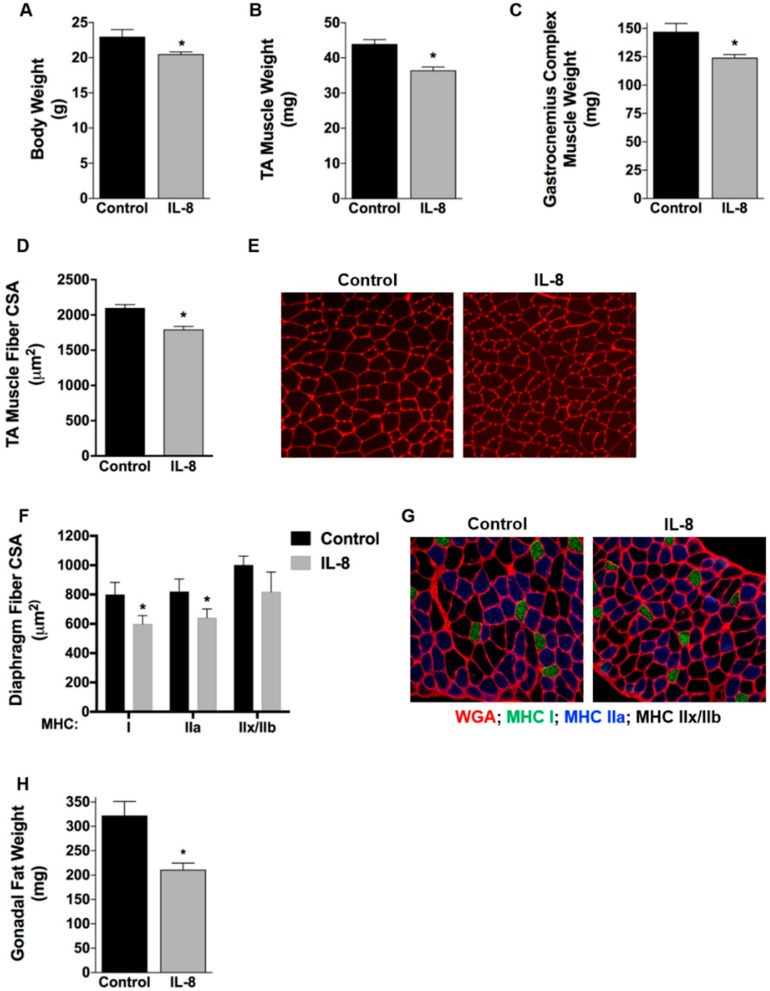
IL-8 induced skeletal muscle and fiber atrophy. (**A**) Body weight, (**B**) tibialis anterior muscle weight, (**C**) gastrocnemius complex muscle weight, and (**H**) gonadal fat weight in mice injected i.p. every other day with 50 μg/kg of IL-8 (*n* = 5 mice) or vehicle (n = 4 mice) for 6 days and tissues harvested 24 h after the last injection. (**D**) Mean TA muscle fiber cross-sectional area measured from wheat germ agglutinin stained cross-sections (**E**). (**G**) Cross-sections cut from the diaphragm muscle and stained with wheat germ agglutinin (red) and immunostained for myosin heavy chain (MHC) type I (green) and MHC type IIa (blue). Unstained (black) fibers are MHC type IIb/x. (**F**) Fiber-type-specific CSA measured in diaphragm muscles. Data presented as mean ± SEM. * *p* < 0.05 compared with control.

To determine whether this IL-8-induced myotube atrophy extends to muscle atrophy in vivo we injected mice intraperitoneally every other day with 50 μg/kg of rIL-8, or vehicle, for 6 days. IL-8-treated mice showed a significant decrease in body weight, and weight of the tibialis anterior (TA) and gastrocnemius muscles (Figure 3A–C). To determine whether this decrease in muscle weight is reflected by a decrease in muscle fiber size, we incubated cross-sections cut from the TA muscle in wheatgerm agglutinin, to outline fiber membranes, and measured the average muscle fiber cross-sectional area (CSA). As shown in Figure 3D,E, TA muscle fiber CSA decreased 15% in IL-8 injected mice (Con: 2099.09 ± 47.07 μm^2^; IL-8: 1795.25 ± 40.69 μm^2^). We similarly cut cross-sections from the diaphragm of mice since this muscle is also significantly atrophied in tumor-bearing mice and is essential for survival [21,22]. Moreover, since the diaphragm is heterogeneous in its fiber type, we conducted immunohistochemistry to identify type I, IIa, and IIb/x myofibers for measurement of fiber type-specific CSA. We found a significant 25% and 22% decrease in the average CSA of type I and IIa fibers, respectively, in IL-8 treated mice compared to controls (Figure 3F,G). The CSA of type IIb/x fibers was also decreased by 18%, but this did not reach statistical significance. These findings demonstrate that IL-8 is sufficient to induce muscle and myofiber atrophy. Interestingly, IL-8 also induced significant fat wasting, with a 34% decrease in gonadal fat mass (Figure 3H). This is relevant to cancer cachexia since fat wasting is commonly observed along with muscle wasting [23]. 

### 2.4. Biological Processes in Skeletal Muscle Regulated by IL-8

**Figure 4:**
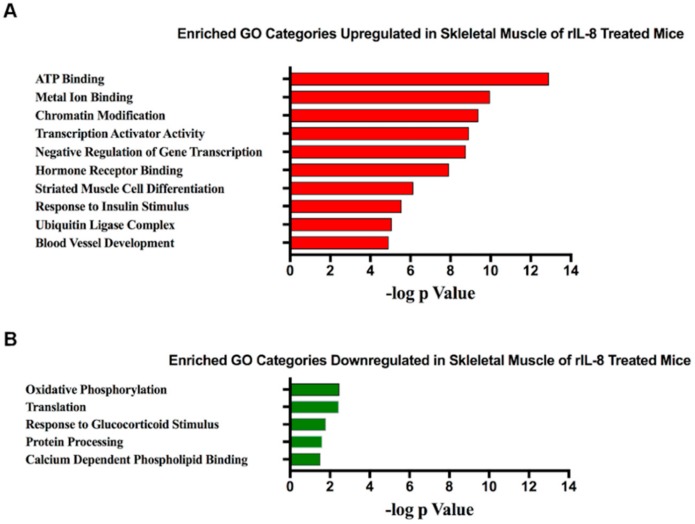
IL-8 responsive skeletal muscle gene networks. Microarray analyses were performed on TA muscles from mice injected i.p. every other day with 50 μg/kg of IL-8 (*N* = 5) or vehicle (*N* = 3) for 6 days and tissues harvested 24 h after the last injection. Enriched Gene Ontology (GO) categories from genes, which were increased (**A**) or decreased (**B**).

Since, to our knowledge, this is the first study to show that IL-8 can induce skeletal muscle wasting in vivo, the biological processes activated in skeletal muscle in response to IL-8 treatment are completely unknown. To address this, we conducted a microarray analysis on TA muscles from vehicle-injected (*N* = 3) and IL-8 injected (*N* = 5) mice and deposited the cel files and expression values into MIAME compliant NCBI Gene Expression Omnibus with accession #GSE137990. We identified 1242 differentially expressed genes (using −1.5 ≥ fold change ≥1.5-fold and q ≤ 0.1) in the muscle of rIL-8 treated mice compared to vehicle-injected mice. Of these 1242 genes, 915 were upregulated and 327 downregulated. These upregulated and downregulated genes were analyzed separately for their associated functional annotations using the DAVID Bioinformatics database [24,25]. The top 10 non-redundant annotation clusters for the upregulated genes, ranked in order of significance, are shown in Figure 4A, which reveal several clusters previously associated with tumor-induced muscle wasting. For example, FoxO1 and FoxO3a were both significantly increased 1.9-fold and annotated to “*transcription activator activity*”. Both FoxO1 and FoxO3a are sufficient to induce skeletal muscle wasting [26,27], and inhibition of FoxO protects against cancer cachexia in mice [22,28]. The category “*ubiquitin ligase complex*” included multiple E3 ubiquitin ligase enzymes, including Fbxo31, Ubr2, and Ubr4. The ubiquitin proteasome pathway is the primary pathway of skeletal muscle protein degradation in tumor-bearing hosts [29], and our finding that IL-8 increases multiple components of this pathway aligns with our finding of IL-8-induced atrophy.

Only five non-redundant annotation clusters were identified for the downregulated genes, which are shown in Figure 4B, ranked in order of significance. Interestingly “*Oxidative Phosphorylation*” was the most enriched GO category, which is highly relevant to pancreatic cancer cachexia. Indeed, recently published microarray data from our lab identified mitochondrial clusters as by far the most enriched clusters for genes downregulated in the skeletal muscle of cachectic pancreatic cancer patients compared to non-cancer controls [30]. 

### 2.5. Interleukin-8 Activates STAT, Smad and ERK Signaling in Myotubes

**Figure 5:**
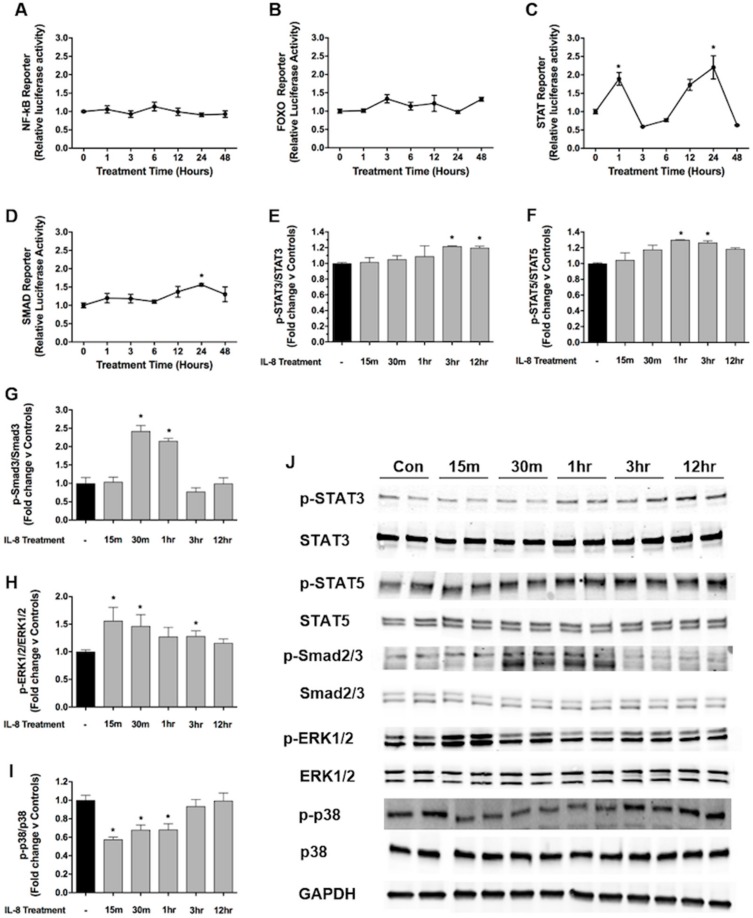
IL-8 responsive signaling pathways in skeletal muscle myotubes. (**A**–**D**) NF-ĸB, FoxO, STAT, and Smad dependent luciferase reporters in C2C12 myotubes treated with 10 ng/ml IL-8 for 1–48 h. STAT3 (**E**), STAT5 (**F**), Smad2/3 (**G**), ERK1/2 (**H**), and p38 (**I**) phosphorylation and total levels in C2C12 myotubes treated with 10 ng/ml IL-8 for 15 minto 12 h. (**J**) Representative Western blots of target proteins and loading control (GAPDH). Data presented as mean ± SEM. * *p* < 0.05 compared with control.

To further explore intracellular signaling pathways activated in skeletal muscle cells in response to IL-8, we transfected C2C12 myoblasts with NF-κB, FoxO, STAT, or Smad dependent luciferase reporter plasmids, differentiated into myotubes and subsequently treated with rIL-8, or BSA, for 1–48 h. These transcription factors were selected because they have been implicated in cancer cachexia [28,31,32,33]. As shown in Figure 5A,B, neither NF-κB nor FoxO dependent reporter activity changes in response to IL-8 treatment across the time frame studied. However, IL-8 induced a biphasic activation of the STAT reporter, with significant increases at 1 and 24 h of treatment (Figure 5C). IL-8 also significantly increased Smad reporter activity at 24 h (Figure 5D). Based on these findings, and the knowledge that STAT and Smad transcription factors are activated via phosphorylation, we measured the phosphorylation and total protein levels of STAT3, STAT5, and Smad2/3 in myotubes treated with IL-8 for 15 minto 12 h. IL-8 treatment caused a significant increase in phospho/total STAT3 at 3 and 12 h (Figure 5E,J) and a significant increase in phospho/total STAT5 at 1 and 3 h of treatment (Figure 5F,J). We also detected a significant increase in phospho/total Smad3 levels at 30 minand 1 hour (Figure 5G,J).

We next measured the phosphorylation and total protein levels of ERK and p38 since both of these MAP kinases are activated in response to IL-8 in neutrophils [34] and cancer cells [35,36,37,38] and have been implicated in tumor-induced skeletal muscle wasting [7,39]. Levels of phospho/total ERK were significantly increased at 15 minutes, 30 minutes, and 3 h of IL-8 treatment (Figure 5H,J), whereas levels of phospho/total p38 were significantly decreased following 15 minutes, 30 minand 1 hour of IL-8 treatment (Figure 5I,J). 

These findings suggest that the exposure of skeletal muscle cells to IL-8 activates several pathways implicated in cancer cachexia. In order to establish the clinical relevance of these in vitro findings, we performed pathway analysis of a recently published microarray from our lab comparing the skeletal muscle transcriptome of cachectic PDAC patients and non-cancer controls [30]. Differentially expressed genes in the skeletal muscle of PDAC patients revealed activated *IL8 Signaling* (z-score = 4.642, *p* < 0.0001), *ERK/MAPK signaling* (z-score = 2.294, *p* < 0.001), *STAT3 pathway* (z-score = 1.886, *p* < 0.0001), and *JAK/STAT signaling* (z-score = 1.291, *p* < 0.0001) (Supplementary Figure S1). 

### 2.6. ERK Inhibition Attenuates Interleukin-8 Induced Skeletal Muscle Wasting

**Figure 6:**
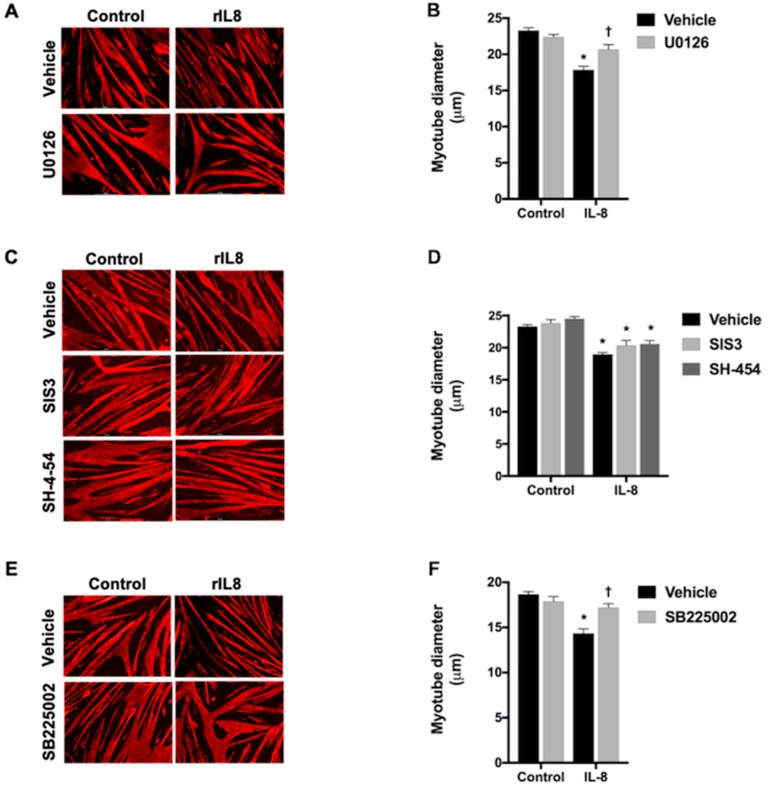
IL-8 signals through CXCR2 and ERK1/2 to induce myotube atrophy. C2C12 myotube representative images (**A**) and diameter (**B**), following 48 h of IL-8 treatment in the presence of the DMSO vehicle or the ERK 1/2 inhibitor UO126. C2C12 myotube representative images (**C**) and diameter (**D**) following 48 h of IL-8 treatment in the presence of either the DMSO vehicle, the Smad3 inhibitor SIS3, or the STAT3/5 inhibitor SH-4-54. C2C12 myotube representative images (**E**) and diameter (**F**), following 48 h of IL-8 treatment in the presence of the DMSO vehicle or the CXCR2 antagonist SB225002. Data presented as mean ± SEM. * *p* < 0.05 compared with control; † *p* < 0.05 compared with the IL-8 vehicle.

Since our findings suggest that IL-8 activates ERK, STAT, and Smad signaling, we next tested whether established inhibitors of each attenuates IL-8-induced myotube atrophy. To inhibit ERK, we treated C2C12 myotubes with U0126, which is well established to inhibit ERK activation by MEK [40]. In this experiment, the significant decrease in myotube diameter in response to IL-8 treatment was statistically abolished in the presence of U0126 (Figure 6A,B), demonstrating that IL-8 induced myotube atrophy is mediated via ERK. To test for a role of Smad3 and STAT3/STAT5 in IL-8 induced myotube atrophy, we treated C2C12 myotubes with SIS3, which is demonstrated to selectively inhibit Smad3 phosphorylation (activation) without inhibiting p38 MAPK, ERK, or PI3K [41,42], or SH-454, which inhibits STAT3 and STAT5 with equal affinity. The IL-8 mediated myotube atrophy was significant across all groups (Figure 6C,D), suggesting that IL-8 mediated myotube wasting does not require Smad3 or STAT3/STAT5 signaling. 

As previously mentioned, the biological effects of IL-8 are mediated through two cell-surface G protein-coupled receptors, CXCR1 and CXCR2, and mice functionally maintain the presence of both receptors [43]. To our knowledge, there are no CXCR1 specific inhibitors, but to test for the requirement of CXCR2 in IL-8-mediated myotube atrophy, we treated myotubes with the CXCR2 antagonist SB 225002, with or without IL-8 for 48 h. As shown in Figure 6E,F, IL-8 treatment induced significant atrophy in vehicle-treated, but not SB 225002-treated, myotubes. This suggests that IL-8-induced myotube atrophy is mediated via the CXCR2 receptor. 

### 2.7. Myotube Atrophy Induced by Human Pancreatic Cancer Cell CM Requires Active Interleukin-8/CXCR2

**Figure 7:**
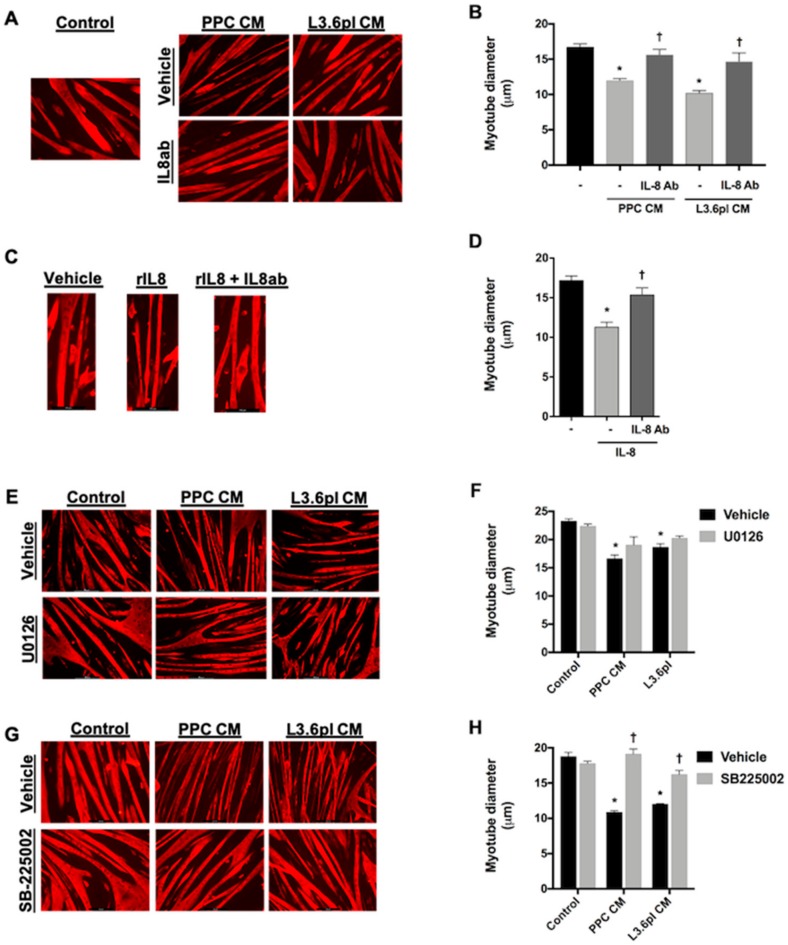
IL-8 is required for myotube atrophy in response to human pancreatic cancer cell CM. C2C12 myotube representative images (**A**) and diameter (**B**), following 48 h of exposure to PPC CM or L3.6pl CM in the presence or absence of an IL8 neutralizing antibody. C2C12 myotube representative images (**C**), and diameter (**D**), following 48 h of IL-8 treatment in the presence or absence of an IL-8 neutralizing antibody. C2C12 myotube representative images (**E**) and diameter (**F**), following 48 h of exposure to PPC CM and L3.6pl CM in the presence or absence of the ERK1/2 inhibitor UO126. C2C12 myotube representative images (**G**) and diameter (**H**), following 48 h of exposure to PPC CM or L3.6pl CM in the presence or absence of the CXCR2 antagonist SB225002. Data presented as mean ± SEM. * *p* < 0.05 compared with untreated control; † *p* < 0.05 compared with the CM vehicle.

To next determine the requirement of active IL-8 for human pancreatic cancer cell CM-induced myotube atrophy, we pretreated CM with a blocking antibody to IL-8 or an isotype control IgG antibody and then added it to 4-day differentiated C2C12 myotubes for 48 h. PPC cell CM induces a 29% decrease in myotube diameter, which was completely prevented by the IL-8 blocking antibody. Similarly, L3.6pl CM induces a significant decrease in myotube diameter, which was inhibited by 84% by the IL-8 blocking antibody (Figure 7A,B). To verify that the IL-8 neutralizing antibody successfully neutralizes IL-8 induced atrophy, we treated myotubes with IL-8 with or without the IL-8 blocking antibody and found complete prevention of atrophy (Figure 7C,D). These findings demonstrate that IL-8 is an active cytokine causing myotube atrophy by human pancreatic cancer cell-conditioned media treatment. 

To next test whether human pancreatic cancer cell CM-induced myotube atrophy, which requires active IL-8, can be attenuated with inhibition of ERK1/2 activation, similar to IL-8 induced atrophy, we treated myotubes with PPC CM or L3.6pl CM, with or without U0126, for 48 h. As shown in Figure 7E,F, PPC and L3.6pl CM induced significant myotube atrophy in the absence, but not presence, of U0126. This suggests that the CM-induced atrophy is mediated, at least in part, via ERK1/2 activation.

Since IL-8 treatment induces myotube atrophy via the receptor CXCR2, we next aimed to determine the extent to which the CXCR2 antagonist, SB225002, could attenuate human pancreatic cancer cell CM-induced atrophy. To do this, we treated myotubes with PPC or L3.6pl CM with or without SB 225002. In this experiment, the significant decrease in myotube diameter in response to PPC CM was completely abolished by SB 225002, while the response to L3.6pl CM was significantly attenuated by 68% (Figure 7G,H). This demonstrates that, similar to IL-8 induced myotube atrophy, myotube atrophy in response to human pancreatic cancer cell CM is mediated through CXCR2. Unfortunately, due to the limited availability of TAS cells we were unable to further determine the requirement of IL-8 for cancer cell/TAS cell CM-induced atrophy.

## 3. Discussion

Pro-inflammatory cytokines are increased in the circulation of tumor-bearing hosts and widely accepted to be a major driver of cachexia. These circulating cytokines derive, in part, from the tumor, and yet our understanding of tumor-derived cytokines which are causative in cachexia is far from complete. In the current study, we conducted multiplex analyte profiling to identify soluble factors released from human pancreatic cancer (PC) cells, which were selected since PC has one of the highest incidences of cachexia [5]. Moreover, since up to 80% of PC tumor mass is tumor-associated stromal (TAS) tissue, we conducted parallel profiling to identify factors released from TAS cells and TAS/PC co-cultures. Of the 41 factors screened, 5 were released from TAS cells at levels >50 pg/mL—EGF (4337 pg/mL), MCP-1 (4951 pg/mL), MCP-3 (110.34 pg/mL), IL-8 (70.94 pg/mL), and IL-6 (70.21 pg/mL), and treatment of myotubes with TAS conditioned media (CM) induced significant atrophy. Thus, TAS cells themselves secrete soluble factors which can drive atrophy of muscle cells in vitro. This is important, as the role that TAS-released factors play in driving cachexia has not been previously studied. 

Our findings that IL-6 and IL-8 are synergistically increased in the conditioned media of PC/TAS cell co-cultures agrees with previously published work from our group [44]. However, here we arrived at this finding through the unbiased screening of 41 cytokines/chemokines, whereas in our previous work, IL-6 and IL-8 were preselected for their study in the tumor microenvironment [44]. In the context of cachexia, IL-6 is a well-studied cytokine. Indeed, elevated serum levels of IL-6 are sufficient to decrease fat mass, and supraphysiological levels of serum IL-6 can also decrease lean mass [45]. Moreover, IL-6 knockout mice bearing Ehrlich carcinoma show significantly less cachexia than wild type controls [46]. However, to our knowledge, there has been no previous mechanistic study of IL-8 as a potential cachexia-inducing factor.

IL-8 is a small 6-8 kDa protein belonging to the cysteine-X-cysteine (CXC) chemokine family [47] and is also known as C–X–C motif ligand 8 (CXCL-8). It was first described as a neutrophil chemoattractant secreted by monocytes and macrophages [48]. However, many other cell types are now known to secrete IL-8, including neutrophils [49], lymphocytes [50], fibroblasts [51], endothelial cells [52], as well as several types of cancer cells [38,44,53,54,55]. In this latter regard, tumor-derived IL-8 can function in an autocrine manner to facilitate oncogenic signaling and pro-metastatic processes, and in a paracrine manner to alter the immune cell populations in the tumor microenvironment and induce angiogenesis [56]. IL-8 is also released from the tumor microenvironment into the systemic circulation, and serum IL-8 levels are increased in patients with a variety of cancers, including pancreatic, colon, renal, prostate, liver, esophageal, and lung cancers [18,19,57,58,59]. Moreover, high serum IL-8 levels are significantly correlated with worse overall survival in patients with pancreatic cancer, hepatocellular carcinoma, renal cell carcinoma, and melanoma. [18,57]. Given that cachexia is also associated with worse overall survival in cancer patients [5,18,60], it is not surprising that IL-8 has been associated with cachexia. Indeed, IL-8 is further increased in the serum of cachectic compared to non-cachectic patients with pancreatic, prostate, and gastroesophageal cancers [18,19,20]. Recent work further showed elevated IL-8 in cachectic versus non-cachectic resected and locally advanced pancreatic cancer patients, which was not the case for IL-6, IL-1β, or TNF-α [18]. This same study also found a significant positive correlation between serum IL-8 levels and weight loss, and a significant negative correlation between serum IL-8 and muscle mass measured from CT scans, in pancreatic cancer patients. Thus, clinical data are highly suggestive of a role for IL-8 in cancer cachexia. 

Given this, it is somewhat surprising that there is a lack of mechanistic studies evaluating the role of IL-8 as a cachexia-inducing factor. This may be because the *Cxcl8* gene, and thus IL-8, is lacking in the mouse genome, and most mechanistic experiments utilize mouse models. However, mice do retain the presence of the two functional IL-8 receptors, CXCR1 and CXCR2 [43,61,62], since other chemokines also signal through these receptors. Indeed, in mice, NAP-2 and LIX both signal through CXCR1, and CXCL1, CXCL2, CXCL3, NAP-2, and LIX all signal through CXCR2 [63]. Moreover, in very recently published work, treatment of C2C12 myotubes with supraphysiological levels (100 and 1000 ng/mL) of recombinant IL-8 was sufficient to induce myotube atrophy [64]. Thus, mouse cells are responsive to human IL-8. In the current study, we show that 10 ng/mL of IL-8 induces comparable atrophy of both mouse C2C12 myotubes and human myotubes. We also found activation of ERK and STAT signaling in C2C12 mouse myotubes in response to human IL-8, which are both IL-8 responsive pathways in human non-muscle cells [65,66]. Thus, although there may be limitations to the the use of mouse cells and tissues to study the role of IL-8, the presence of the IL-8 receptors on mouse cells, and their functional responsiveness to IL-8, suggests they can provide a suitable system in which to study IL-8. Indeed, a multitude of studies have previously explored the role of IL-8 using mouse cells or mice [61,62,64,67,68,69,70].

Our finding that treatment of mice with IL-8 causes significant skeletal muscle wasting identifies IL-8 as an atrophy-inducing chemokine. Although this muscle atrophy in mice could be due to direct or indirect effects of IL-8 on muscle, our finding that IL-8 induces atrophy of myotubes, in vitro, demonstrates that IL-8 can act directly on muscle cells. This IL-8-induced atrophy appears to be mediated through CXCR2, since treatment with the CXCR2 antagonist blocks the IL-8-induced atrophy. Thus, although we did not test for a role of CXCR1, due to the lack of any specific inhibitors, the lack of significant atrophy in response to IL-8 when CXCR2 is selectively inhibited suggests a minimal, or no, role of CXCR1. 

As already mentioned, the binding of IL-8 to CXCR2 in non-muscle cells is known to activate STAT and ERK signaling [65,66], but also to activate NF-κB [71] and p38 MAPK signaling [65], and each of these signaling pathways is implicated in tumor-induced skeletal muscle atrophy [7,31,32,39]. Thus, we hypothesize that IL-8 may induce atrophy of muscle by activating one or more of these signaling pathways. From our survey of these pathways, we found IL-8 activates STAT and ERK1/2 signaling, induces no changes in κB signaling, and represses p38 MAPK. We also selected to measure FoxO and Smad signaling, not because they have been shown to be IL-8-responsive transcription factors, but because these pathways have also been implicated in tumor-induced muscle atrophy [28,33]. We found that IL-8 activates the Smad reporter and increases phospho(active)-Smad3 but induces no change in FoxO reporter activity. Although these combined findings suggest that IL-8-induced muscle atrophy may proceed through STAT, ERK, and/or Smad signaling, our data show that only inhibition of ERK1/2 blocks IL-8 induced myotube atrophy. This requirement of ERK1/2 is consistent with its requirement for colon adenocarcinoma (C26) conditioned media induced myotube atrophy and leukemia inhibitory factor-induced myotube atrophy [72], as well as cachexia in C26 tumor-bearing mice [39]. Thus, IL-8 induced myotube atrophy requires a CXCR2-ERK1/2 signaling axis. Although we did not determine the downstream targets of this axis in myotubes, our in vivo microarray data show that several genes annotating to the ubiquitin proteasome pathway, including *Fbxo31*, *Ubr2*, *Ubr4*, *Ubr5*, *Ube3b*, *Ubqln1*, *Ubqln4*, *Psmd3*, and *Fbxl17* are increased in skeletal muscle in response to IL-8, as are the autophagy-related genes *Ulk1* and *Ulk2*. These genes may, therefore, be downstream targets of this IL-8-CXCR2-ERK1/2 axis in skeletal muscle. Our subsequent findings that IL-8 neutralization or inhibition of CXCR2 prevents myotube atrophy in response to PPC CM and L3.6pl CM identifies IL-8 as the major soluble mediator in PC CM which causes myotube atrophy. Furthermore, since inhibition of ERK1/2 prevents significant myotube atrophy in response to PPC CM and L3.6pl CM, we can conclude that myotube atrophy in response to human PC cell-conditioned media requires the IL-8-CXCR2-ERK1/2 axis. 

## 4. Materials and Methods

### 4.1. Cells

The primary human pancreatic cancer (PPC) cell line was previously isolated, and methods described [73]. Briefly, a 2 × 2 mm viable portion of resected pancreatic tumor tissue, obtained with informed written patient consent and approved by the University of Florida Institutional Review Board, was implanted subcutaneously into immunocompromised mice and allowed to grow to 1.5 cm. The xenograft tumor was removed, minced and enzymatically digested, seeded onto collagen-coated plates, and cultured in advanced Dulbecco’s Modified Eagle Medium DMEM; Thermo Fisher Scientific, Waltham, MA, USA) with nutrient mixture F12, 10% fetal bovine serum (FBS; Thermo Fisher Scientific), 6 mmol/L glutamine, 1% of penicillin/streptomycin, and 40 ng/mL dexamethasone (Thermo Fisher Scientific). Cells were differentially trypsinized to remove any contaminating fibroblasts, and homogeneity confirmed with cytokeratin 19 and class I human leukocyte antigen (HLA) markers. 

Human L3.6pl pancreatic cancer cells were originally derived from repeated in vivo injections of the COLO-357 pancreatic cell line, as previously described [74,75], and maintained in DMEM, 10% FBS, and 1% penicillin/streptomycin. The primary human pancreatic tumor-associated stroma (TAS) line was previously isolated and described by Han et al. [76] from freshly resected pancreatic surgical specimens. The PPC and TAS cell lines were maintained in advanced DMEM/F12 containing 1X GlutaMAX (Thermo Fisher Scientific), 100 ng/mL recombinant human epidermal growth factor (Sigma-Aldrich, St. Louis, MO, USA), 10% FBS, and penicillin/streptomycin, and incubated at 37 °C in 5% CO_2_.

Murine C212 myoblasts were obtained from the American Type Culture Collection (Manassas, VA, USA) and cultured in DMEM, 10% FBS, and 1% penicillin/streptomycin at 37 °C in 5% CO_2_. Myoblasts were differentiated in DMEM containing 2% horse serum (Thermo Fisher Scientific), and 1% penicillin/streptomycin. The differentiation medium was changed every other day. Human skeletal myoblasts were obtained from Cook MyoSite (Pittsburgh, PA, USA), and cultured using the Cook Myosite MyoTonic™ media kit with 10% FBS according to the manufacturer’s instructions. The human skeletal muscle-derived cells were differentiated using MyoTonic™ differentiation medium.

### 4.2. Conditioned Media

Conditioned media (CM) was collected by plating 1x10^6^ PPC, L3.6pl or TAS cells in a T-25 cell culture flask. CM from co-culture experiments was generated from L3.6pl:TAS co-cultures and PPC:TAS co-cultures plated in a 1:1 ratio. The following day cells were washed twice with 1X phosphate buffered saline (PBS) (Thermo Fisher Scientific) and once with appropriate serum-free growth media containing antibiotics. Cells were then incubated for 24 h in fresh serum-free media. CM was collected from the culture flask, centrifuged at 2000× *g* for 15 minutes, and then filtered through 0.22 µm. Aliquots of CM were stored at −80 °C and used for all experiments. For crosstalk experiments, cancer or stromal cells were plated at a density of 2 × 10^5^ in 12-well plates and allowed to rest overnight. The indicated CM was added at 10% or 50% in appropriate growth media, and the final conditioned media collected at 24 h, centrifuged, filtered, and stored. 

### 4.3. Cytokine and Chemokine Analysis

Conditioned media was screened for soluble mediators using the Premixed 41-Plex human cytokine/chemokine magnetic bead panel (MilliporeSigma, Burlington, MA, USA) according to the manufacturer’s instructions. Data were acquired on a Luminex^®^ 200 (Luminex Corp, Austin, TX, USA), and cytokine and chemokine concentrations were determined based on a five-parameter logistic spline-curve fitting method using the MILLIPLEX^®^ Analyst 5.1 software (Vigene Tech Inc., Carlisle, MA, USA). The analytes tested were sCD40L, EGF, FGF-2, Flt-3 ligand, Fractalkine, G-CSF, GM-CSF, GRO, IFN-α2, IFNγ, IL-1a, IL-1b, IL-1ra, IL-2, IL-3, IL-4, IL-5, IL-6, IL-7, IL-8, IL-9, IL-10, IL-12 (p40), IL-12 (p70), IL-13, IL-15, IL-17A, IP-10, MCP-1, MCP-3, MDC (CCL22), MIP-1α, MIP-1β, PDGF-AB/BB, RANTES, TGF-α, TNF-α, VEGF, Eotaxin/CCL11, PDGF-AA.

### 4.4. Myotube Treatments and Diameter Measurements

Murine C2C12 myoblasts were differentiated and treated on day 4 with 10ng/mL of recombinant human IL-8 (R&D Systems, Minneapolis, MN, USA), or conditioned media in a ratio of 1:3 with fresh differentiation media, for 48 h. To neutralize IL-8, CM was incubated with 1µg/mL of a human IL-8 antibody (R&D Systems, Minneapolis, MN, USA) for 1 hour at 37 °C prior to the addition of CM to myotubes. Mouse IgG_1_ was used at the same concentration as an isotype control. To inhibit ERK1/2, STAT3/5, Smad3, and CXCR2, differentiated myotubes were treated with 10 ng/mL of recombinant IL-8 in the presence of the following inhibitors and concentrations: U0126, 1 uM (Tocris Bioscience, Bristol, UK); SIS3, 3uM (MilliporeSigma); SH-4-54, 2uM (MilliporeSigma); and SB225002, 22nM (R&D Systems, Minneapolis, MN, USA); or with a DMSO vehicle control. In experiments where PPC and L3.6pl CM were pretreated with inhibitors U0126 and SB225002, CM was treated at a concentration of 1 uM and 22 nM, respectively. for 12 h. Myotubes were then fixed in 2% paraformaldehyde (Boston BioProducts, Ashland, MA, USA) for 30 minutes, permeabilized, and blocked for 30 minin 3% bovine serum albumin (BSA) (Sigma-Aldrich) with 0.5% triton X-100 (Thermofisher) in 1X PBS. Myotubes were incubated with mouse monoclonal anti-myosin heavy chain antibody MF20 (Developmental Studies Hybridoma Bank, Iowa City, IA) overnight at 4 °C. The cells were then washed with 1X PBS and myotubes stained with Rhodamine Red-X goat anti-mouse IgG (Thermofisher Scientific) for 1 hour at room temperature. Myotubes were imaged on a Leica DMI3000 B inverted microscope, equipped with an N2.1 filter set (Leica Microsystems, Buffalo Grove, IL, USA). Myotube diameter was measured using ImageJ (https://imagej.nih.gov/ij/), where hand-traced diameters of at least 2 measurements per myotube were made. A minimum of 150 myotubes was measured per group. Images are acquired at 20X unless otherwise stated. 

### 4.5. Plasmids

The NF-κB reporter plasmid was obtained from Dr. Steffan Ho and the FoxO reporter from Dr. Alex Toker (Beth Israel Deaconess Medical Center, Boston, MA, USA), and both have been previously used and described [77,78]. The Smad (CAGA_12_-luciferase reporter) was obtained from Dr. Peter Ten Dijke (Leiden University Medical Centre, Leiden, The Netherlands), and has previously been used and described [79]. The STAT reporter plasmid was obtained from Addgene (Watertown, MA, USA; plasmid 8688), where it was deposited by Jim Darnell and has previously been described [80]. pRL-TK-*Renilla* was purchased from Promega (Madison, WI, USA).

### 4.6. Luciferase Reporter Assays

C2C12 myoblasts were transiently transfected in 12-well plates with firefly luciferase reporter plasmids using FuGENE^®^ HD (Promega, Madison, WI, USA) according to the manufacturer’s instructions. Each experimental condition was co-transfected with a *Renilla* luciferase reporter. After transfection, myoblasts were differentiated for 4 days and then myotubes were treated with 10ng/mL of recombinant IL-8 for the indicated time points. Cells were lysed in passive lysis buffer (Promega) with protease and phosphatase inhibitors (MilliporeSigma) and transferred to microcentrifuge tubes. Cell lysates were centrifuged at 9500× *g* for 10 minat 4 °C and supernatant was collected and mixed with luciferase assay reagent (Promega) according to the manufacturer’s instructions. Luciferase activity was measured with a Turner Biosystems modulus luminometer (Promega), and firefly luciferase activity was normalized to *Renilla* luciferase activity.

### 4.7. Western Blots

Four-day differentiated C2C12 myotubes were treated at the time points indicated, washed twice in 1X PBS (Thermo Fisher Scientific), and lysed in radioimmunoprecipitation assay (RIPA) buffer (MilliporeSigma) on ice for 30 minutes. Cells were then scraped into 1.7mL Eppendorf tubes, centrifuged at 9000× *g* for 10 minat 4 °C, and supernatant collected. Protein lysates were quantified by BCA assay (Thermo Fisher Scientific) according to the manufacturer’s instructions. Equal amounts of protein were separated by SDS-PAGE (4%–15% TGX stain-free gel Bio-Rad, Hercules, CA, USA), and proteins transferred using the trans-blot turbo transfer system (Bio-Rad). The nitrocellulose membranes were probed with the following antibodies from Cell Signaling (Danvers, MA, USA), according to established Western blot protocols: p-STAT3 (#9145), STAT3 (#9139), p-STAT5 (#9351), STAT5 (#94205), p-SMAD2 (#3104), p-SMAD3 (#9520), SMAD2/3 (#8685), p-ERK1/2 (#4370), ERK1/2 (#4695), IκBα (#4814), p-p38 (#9216), p38 (#9212), and GAPDH (#2118). The secondary signal was quantified by fluorescence using a LI-COR Odyssey^®^ imager (LI-COR Biosciences, Lincoln, NE, USA), and the signal was normalized to a GAPDH loading control.

### 4.8. Animals

All animal studies were approved by the University of Florida Institutional Animal Care and Use Committee (Animal Welfare Assurance Number A3377-01, protocol 201608146, approved on 17 November 2016) and were in compliance with the National Institutes of Health Guidelines for Use and Care of Laboratory Animals. Mice were housed at the University of Florida animal facility in a regulated temperature and humidity environment under a 12-hour light/dark cycle. Animals were provided standard diet and water ad libitum. 

### 4.9. IL-8 Treatment of Mice

Eight-week-old male C57BL/6J mice were purchased from The Jackson Laboratory (Bar Harbor, ME, USA), and 50 µg/kg recombinant human IL-8 (R&D Systems), diluted in 0.1% BSA, was administered by intraperitoneal injection every other day for 6 days. Age-matched control animals were given 0.1% BSA as control injections. Animals were euthanized on day 7 and tibialis anterior (TA), gastrocnemius complex, diaphragm, gonadal fat, and peripheral blood taken. Muscles were rinsed in 1X PBS, weighed, and flash-frozen in liquid nitrogen for RNA isolation or embedded in OCT and frozen in liquid nitrogen-cooled isopentane for cryosectioning. 

### 4.10. Muscle Immunohistochemistry and Measurements

Tibialis anterior muscles were cut across the midbelly and 10 µm sections transferred onto glass slides using a Microm HM 550 Cryostat (Microm International, Walldorf, Gemany). Tissue sections were fixed with 4% paraformaldehyde for ten minutes, then incubated with wheat germ agglutinin conjugated to Alexa Fluor™ 594 (Thermo Fisher Scientific) for 1 hour at room temperature. Whole diaphragm muscles were cryosectioned and fixed as above and stained for myosin heavy chain type I (clone BA-D5, Developmental Studies Hybridoma Bank), myosin heavy chain type IIa (clone SC-71, Developmental Studies Hybridoma Bank) and wheat germ agglutinin conjugated to Alexa Fluor™ 594 (Thermo Fisher Scientific). Secondary antibodies used for MHC type I and MHC type IIa were Alexa Fluor™ 350 and 488, respectively. TA and diaphragm sections were imaged on a Leica DM5000 B upright microscope, and muscle fiber cross-sectional area was measured using ImageJ. A minimum of 250 fibers was measured per muscle.

### 4.11. Microarray and Gene Expression Analysis

Whole TA muscle was homogenized using a polytron homogenizer, and total RNA isolated using TRIzol™ Reagent (Thermo Fisher Scientific) as previously described [81]. RNA purity and concentration in samples was determined by absorbance spectrophotometry. Samples were then shipped to Boston University Microarray and Sequencing Resource Core facility where further RNA quality testing was conducted by Bioanalyzer 2100 analysis (Agilent, Palo Alto, CA, USA). Samples used for microarray had a minimal RIN number of 9.0. Microarray analysis was performed using GeneChip mouse gene 2.0 ST array, as described previously [22]. Differential gene expression analyses (using −1.5 ≥ fold change ≥ 1.5-fold and q ≤ 0.1) were performed in our lab using the comparative marker selection module within GenePattern (Broad Institute) and Gene Ontology (GO) biological processes determined using the DAVID Bioinformatics database [24,25] version 6.8. Recently, our lab performed a microarray analysis of skeletal muscle samples from PDAC patients and non-cancer controls (GSE: 130563) [30]. Bioinformatic analyses of differentially expressed genes (−1.2 ≤ fold change ≥ 1.2) between PDAC patients and non-cancer controls were performed through ingenuity pathway analysis [82].

### 4.12. Statistical Analysis

Statistical analyses were performed using GraphPad Prism^®^ (GraphPad Software, San Diego, CA, USA), and the level for statistical significance was set to *p* < 0.05. Unpaired *t*-tests were conducted for all two-group comparisons, after first testing for normality with Shapiro–Wilk. When more than two groups were compared, an analysis of variance (ANOVA) was used, followed by Bonferroni post hoc comparisons. Data are presented as mean ± SEM.

## 5. Conclusions

In summary, through this body of work, we identified IL-8 as a cachexia-inducing factor in vitro and in vivo that is synergistically released from the interaction of primary human pancreatic cancer cells and tumor-associated stromal cells—which more closely recapitulates the pancreatic tumor microenvironment, in vivo. Mechanistically, we identified that IL8 induces muscle wasting through the engagement of the CXCR2 receptor and activation of ERK signaling and that this signaling axis is necessary for muscle atrophy induced in response to pancreatic cancer cell-conditioned media.

## Supplementary Materials

The following are available online at https://www.mdpi.com/2072-6694/11/12/1863/s1, Table S1: Concentration of cytokines and chemokines in the conditioned medium of human pancreatic cancer and tumor associated stromal cells, and their co-culture, Figure S1: IL8-related canonical pathways enriched in the transcriptome of PDAC patient skeletal muscle.
