# Supplementary material for: IL-8 Released from Human Pancreatic Cancer and Tumor-Associated Stromal Cells Signals through a CXCR2-ERK1/2 Axis to Induce Muscle Atrophy
Source: Cancers (Basel). 2019 Nov 25;11(12):1863. doi: 10.3390/cancers11121863 (PMC6966692; doi:10.3390/cancers11121863)
Supplement: Supplementary file 1 [file cancers-11-01863-s001.zip › Final CANCERS submission supplemental/Supplemental Figure 1 FINAL.pdf]

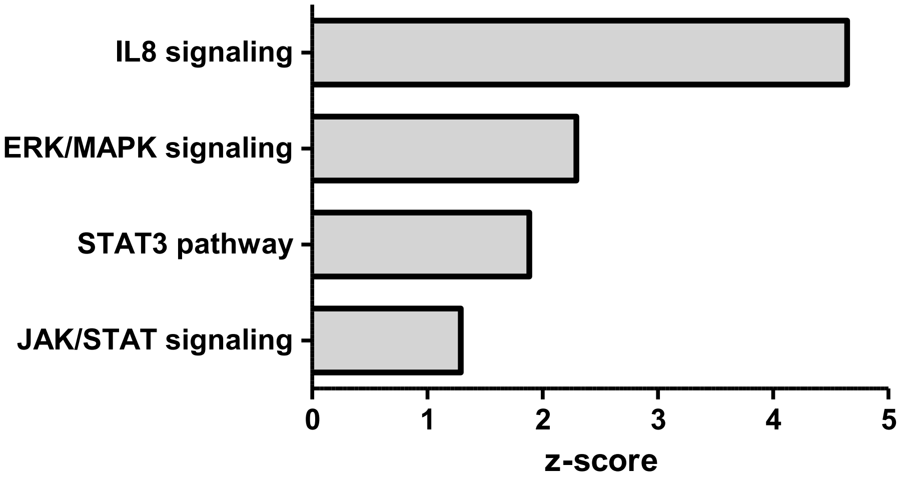

Supplemental Figure 1. IL8-related canonical pathways enriched in the skeletal muscle transcriptome of PDAC patients. Ingenuity Pathway Analysis of a recently published microarray from our lab [30] comparing the skeletal muscle transcriptome of PDAC patients and non-cancer controls reveals significant activation of *IL8 Signaling* (z-score = 4.642, P < 0.0001) in cachectic PDAC patients. In addition, activated pathways identified in IL8-treated C2C12 myotubes also appear to be activated in the skeletal muscle of cachectic PDAC patients. These pathways include *ERK/MAPK signaling* (z-score = 2.294, P < 0.001), *STAT3 pathway* (z-score = 1.886, P < 0.0001), and *JAK/STAT signaling* (z-score = 1.291, P < 0.0001).
