# Supplementary figures and images for: IL-8 Released from Human Pancreatic Cancer and Tumor-Associated Stromal Cells Signals through a CXCR2-ERK1/2 Axis to Induce Muscle Atrophy
Source: Cancers (Basel). 2019 Nov 25;11(12):1863. doi: 10.3390/cancers11121863 (PMC6966692; doi:10.3390/cancers11121863)

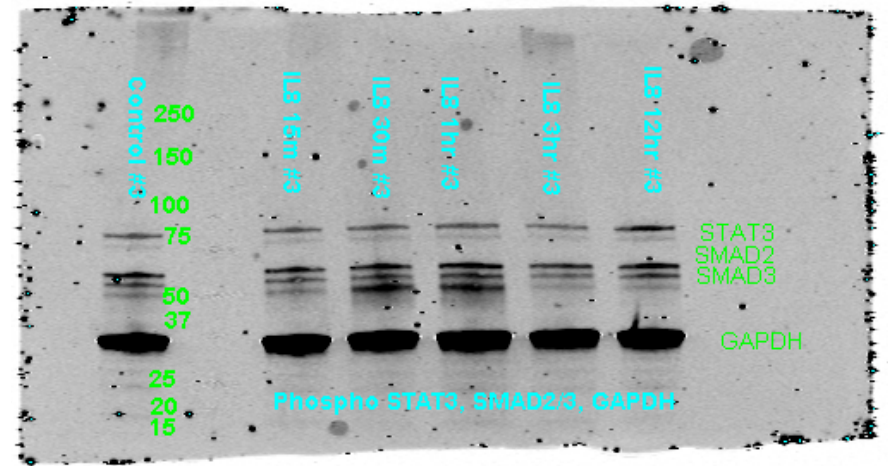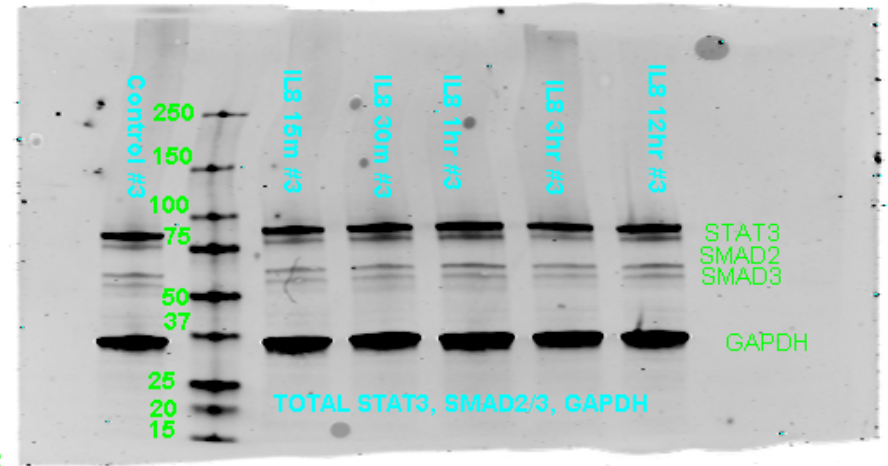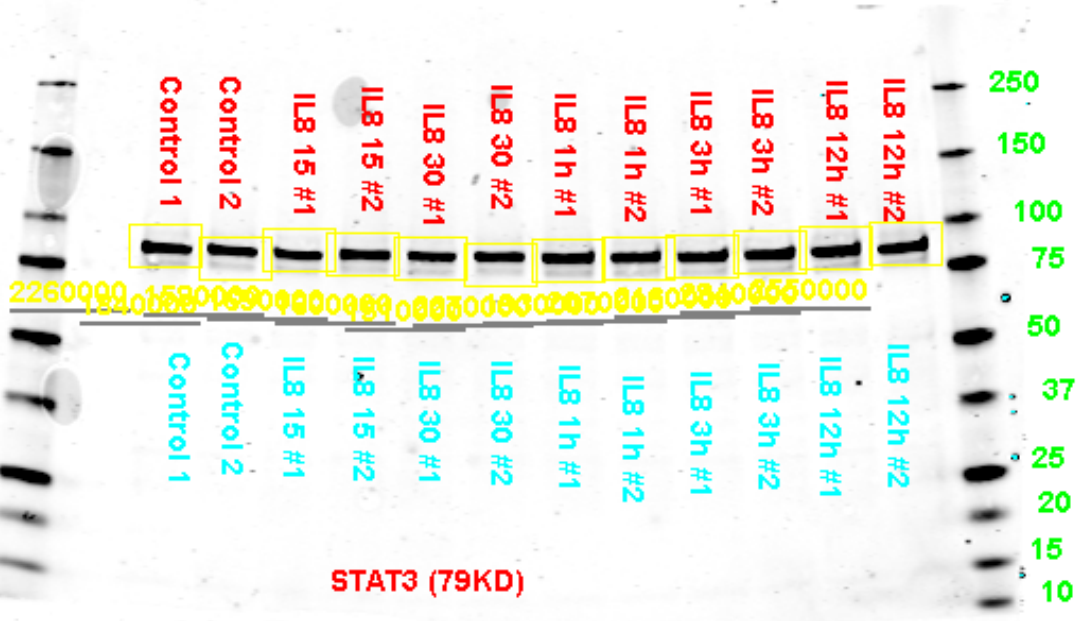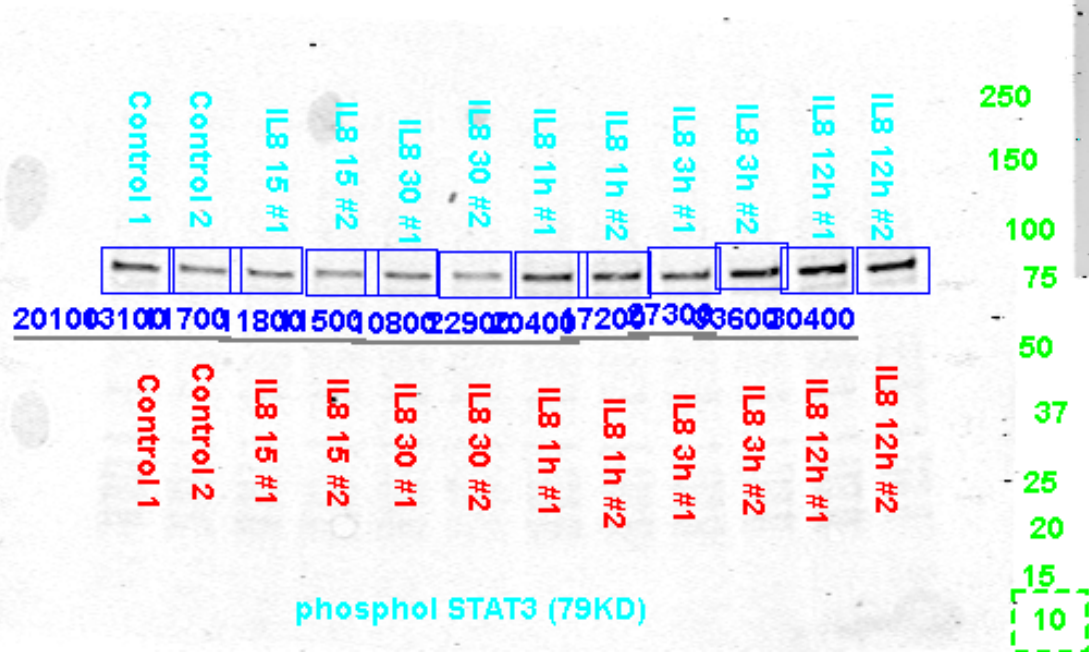

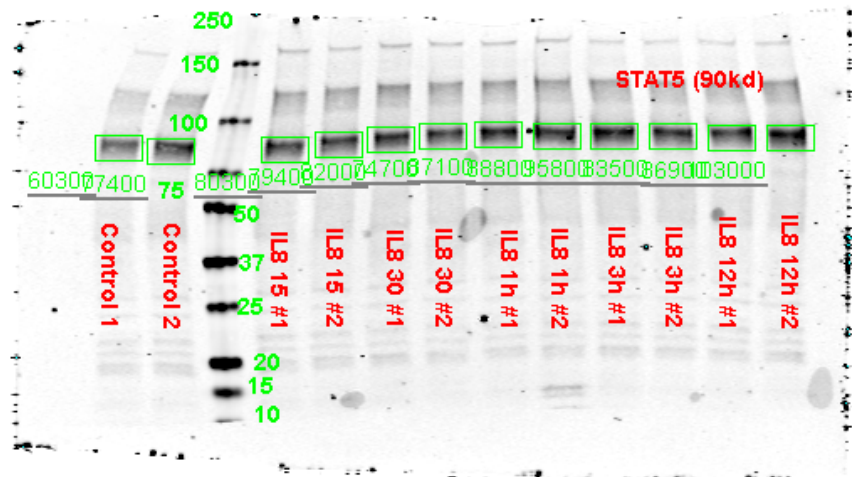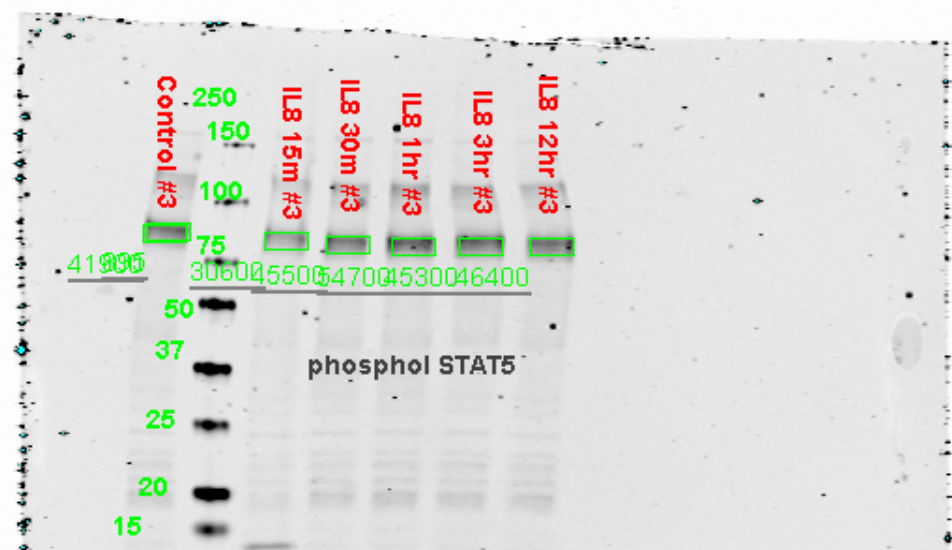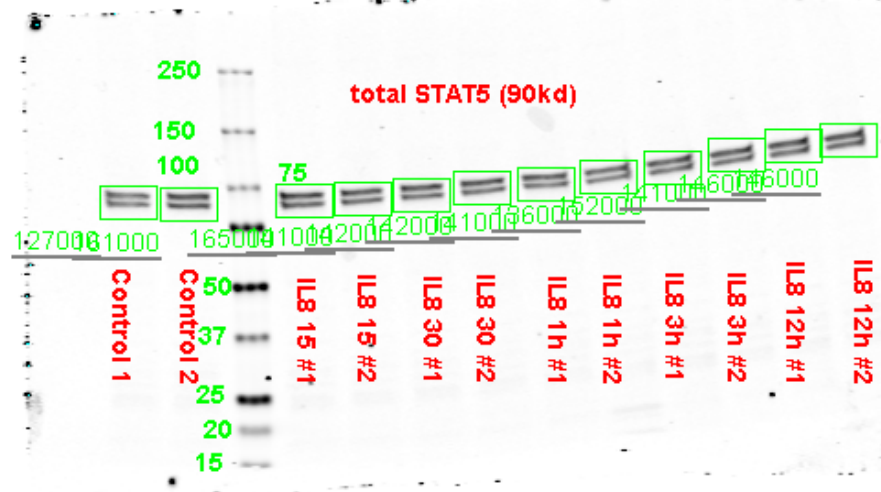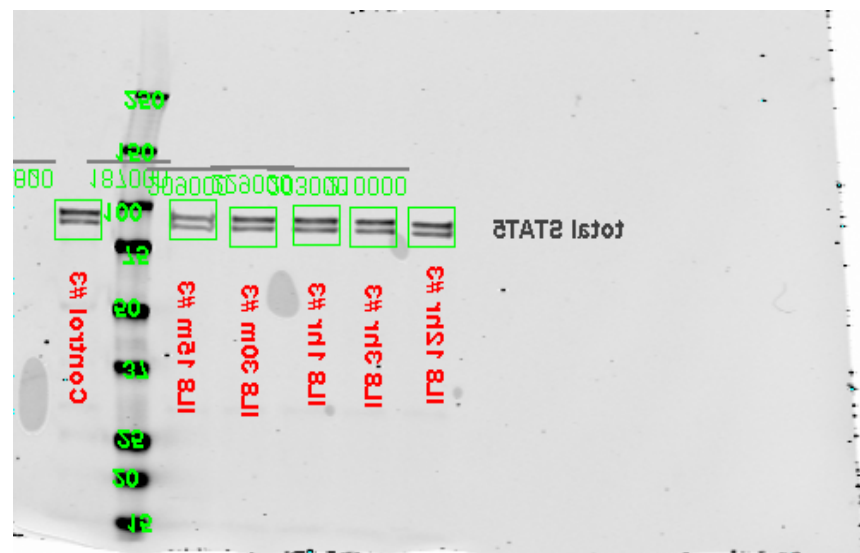

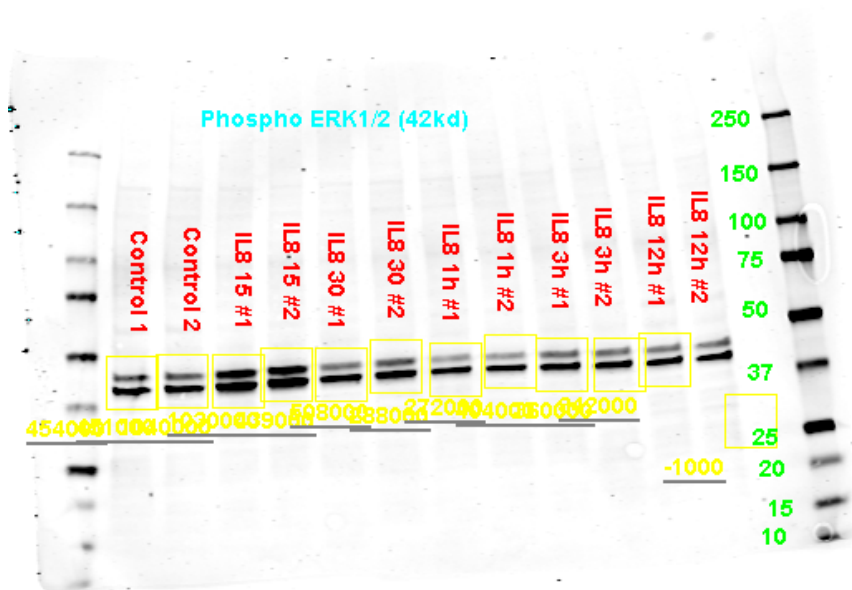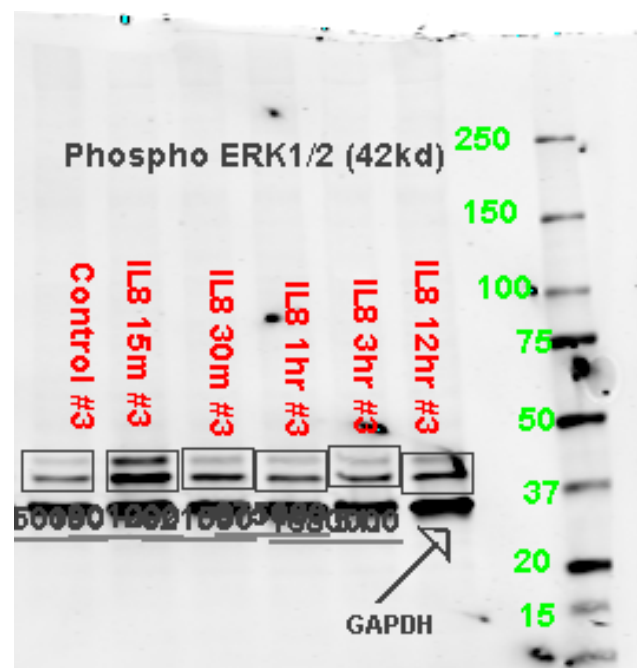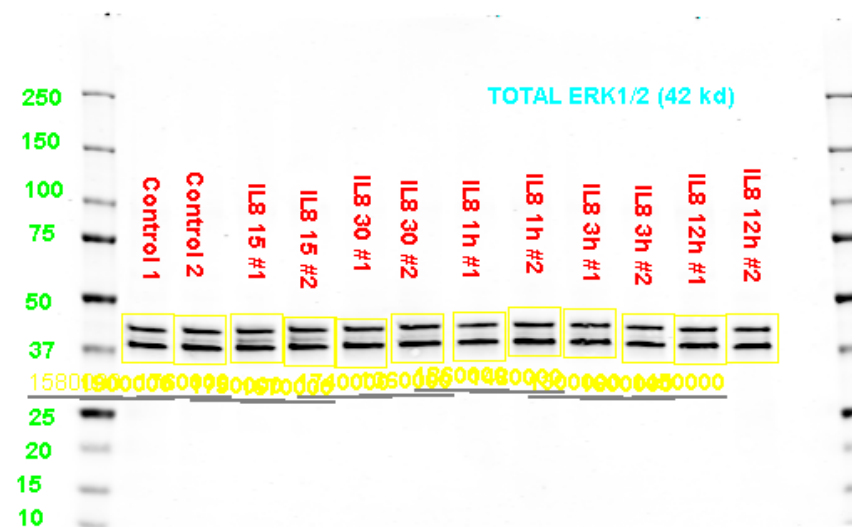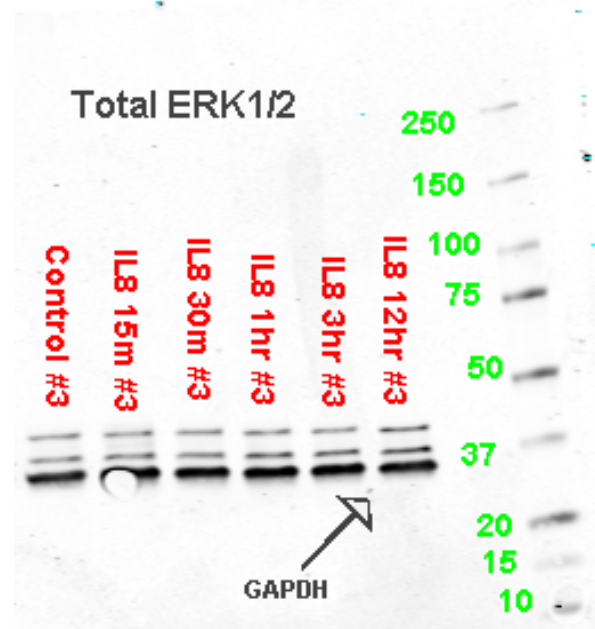

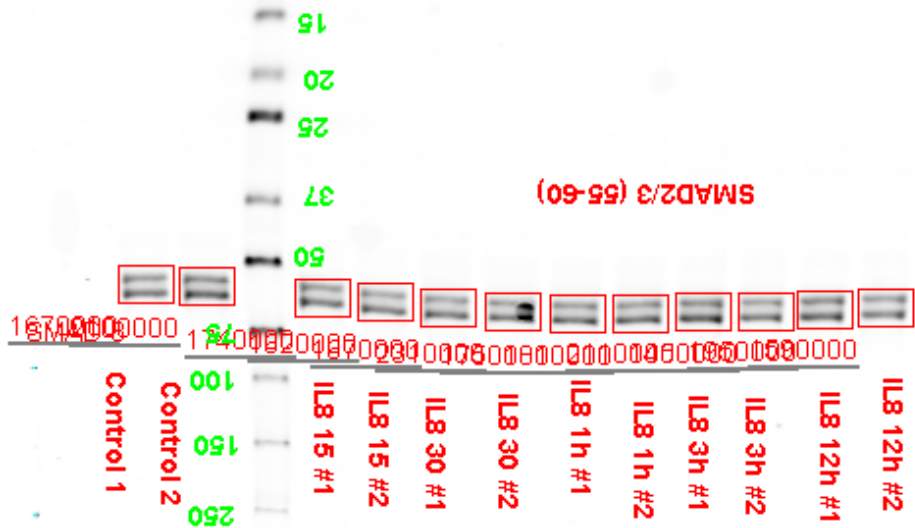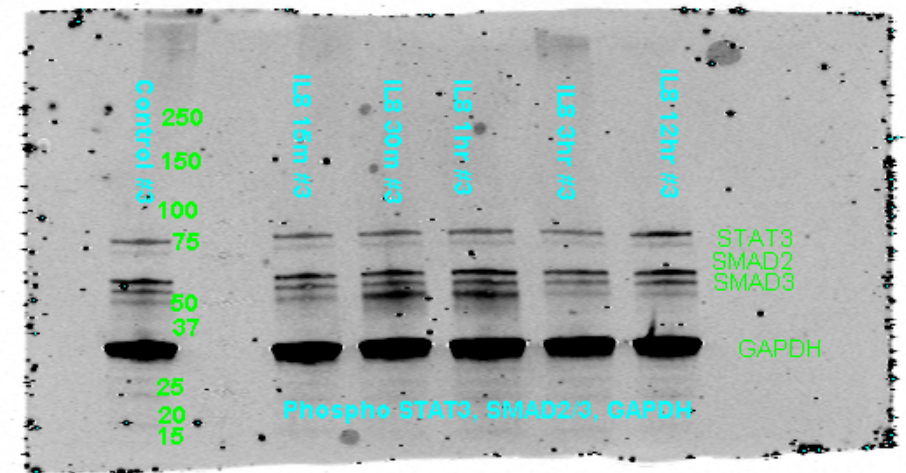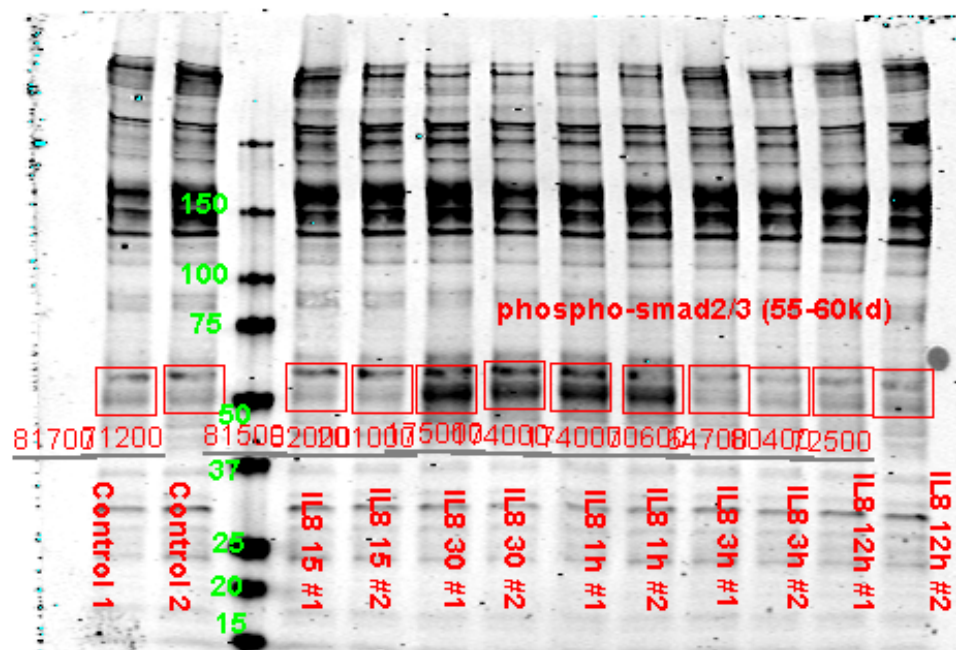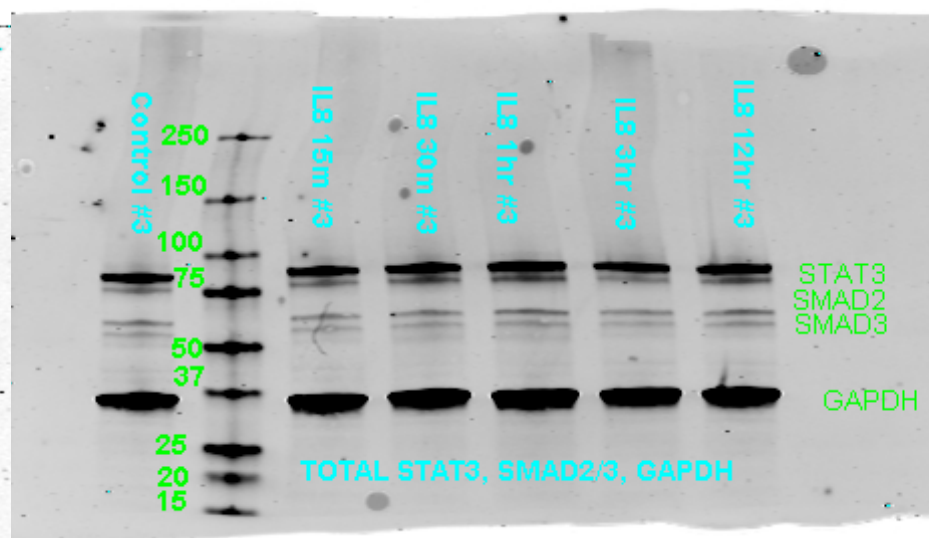

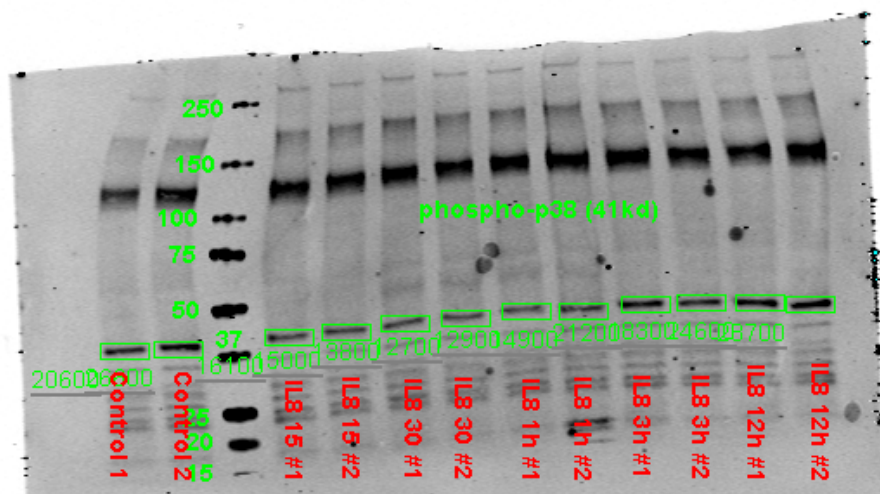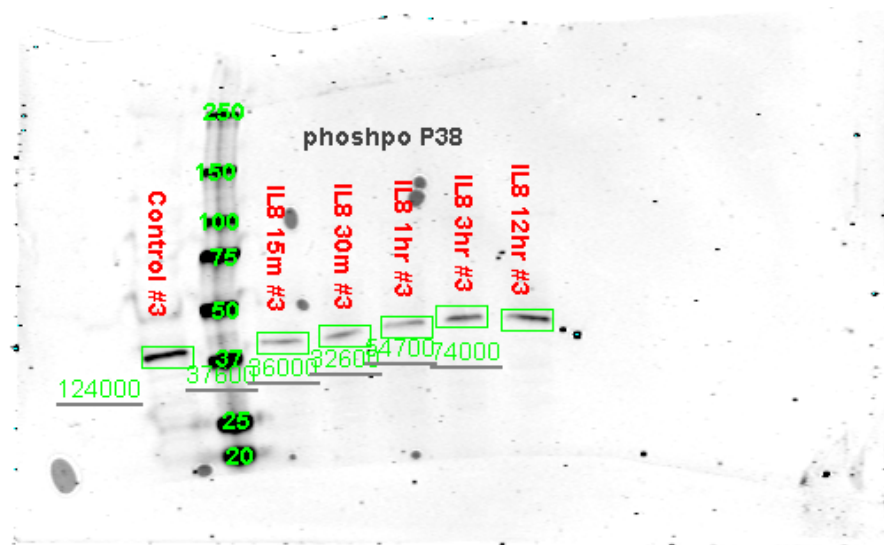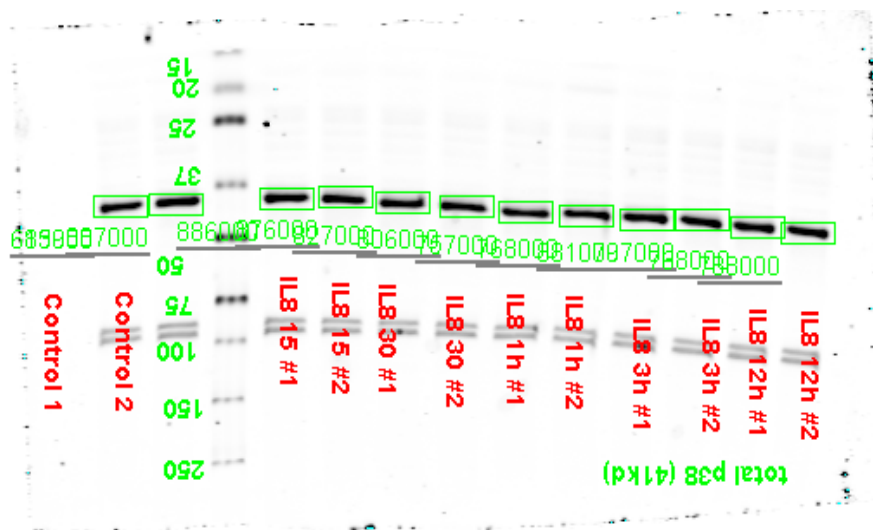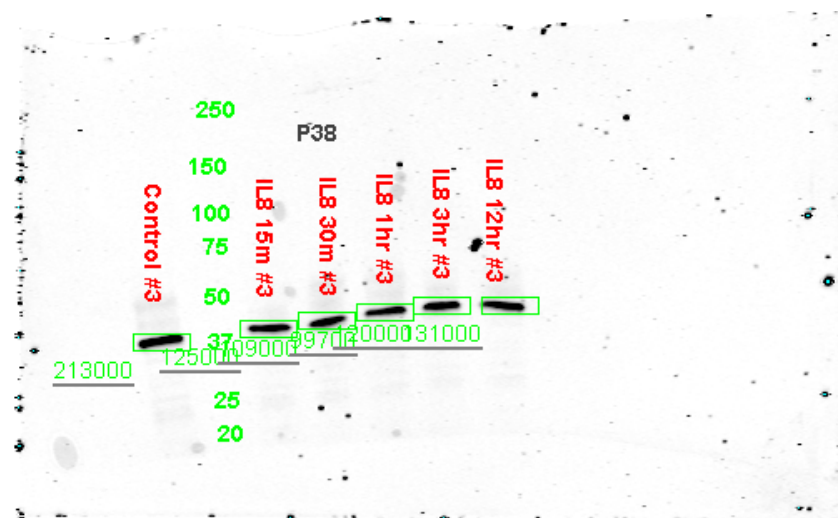

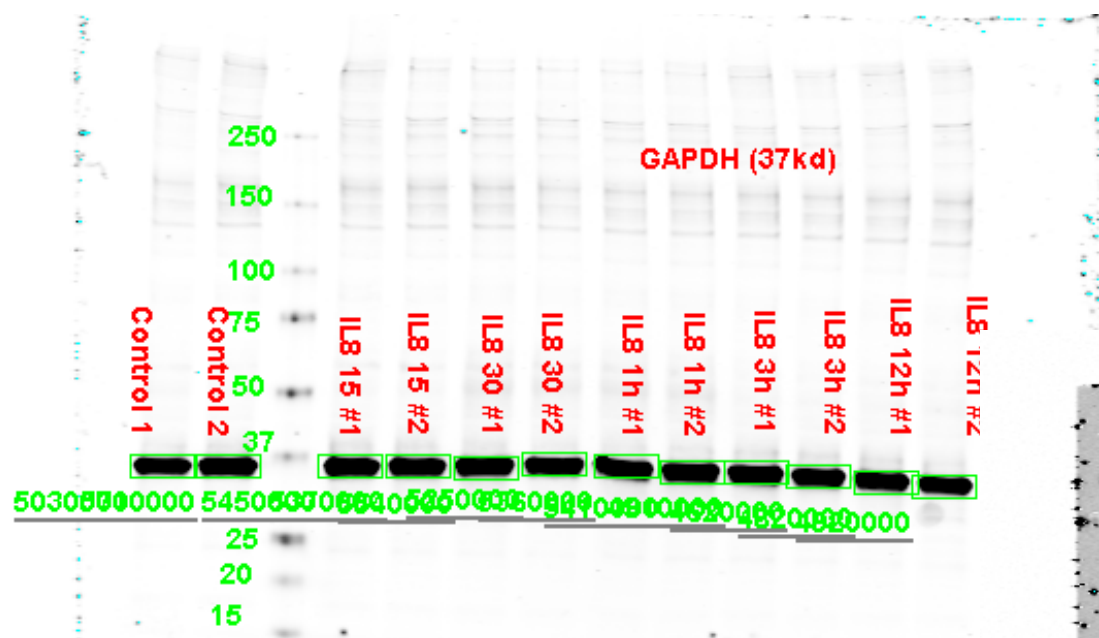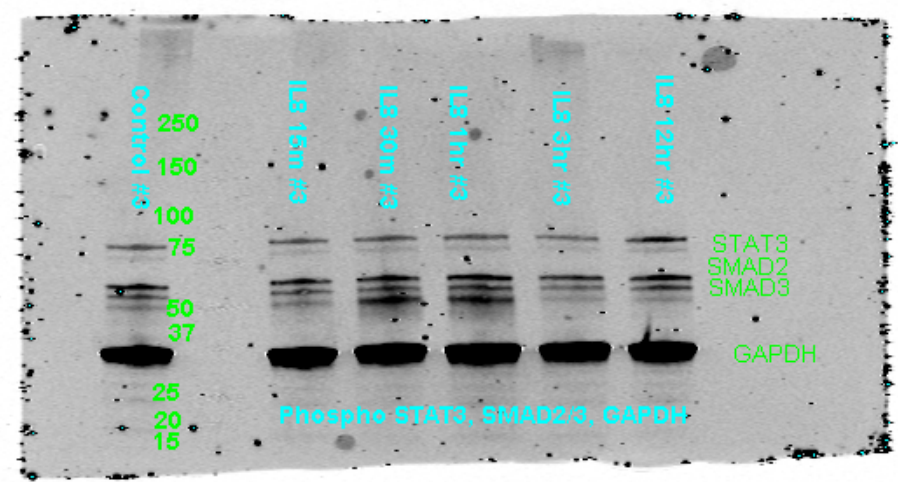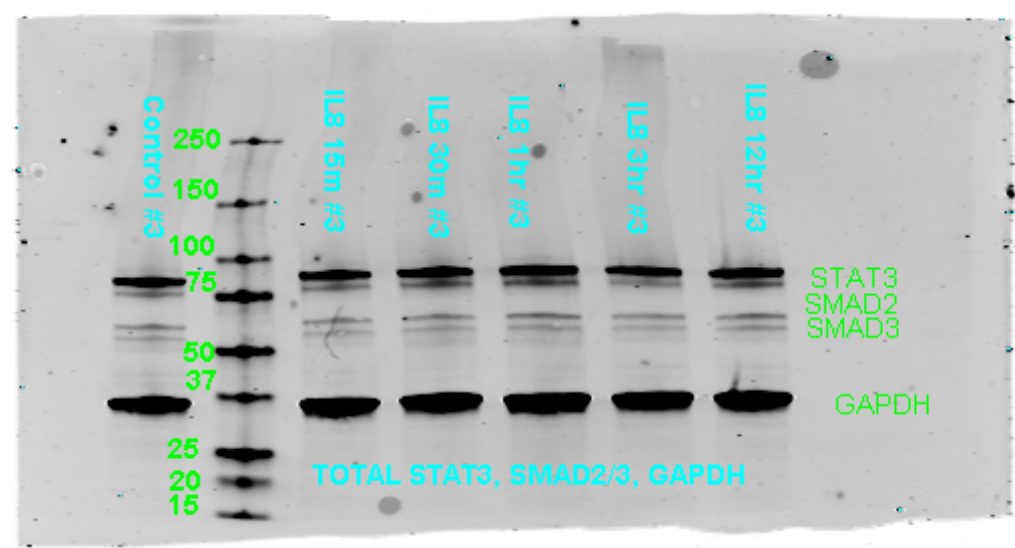

Supplement: Supplementary file 1 [file cancers-11-01863-s001.zip › Western Blot Cancers Submission IL8.pdf]
